# ATLAS: a large array, on-chip compute SPAD camera for multispeckle diffuse correlation spectroscopy

**DOI:** 10.1364/BOE.531416

**Published:** 2024-10-24

**Authors:** Alistair Gorman, Neil Finlayson, Ahmet T. Erdogan, Lars Fisher, Yining Wang, Francescopaolo Mattioli Della Rocca, Hanning Mai, Edbert J. Sie, Francesco Marsili, Robert K. Henderson

**Affiliations:** 1 Institute for Integrated Micro and Nano Systems, School of Engineering, University of Edinburgh, Edinburgh, UK; 2 Currently with Sony Europe Technology Development Centre, 38123 Trento, Italy; 3 Reality Labs, Meta Platforms Inc., Menlo Park, CA 94025, USA

## Abstract

We present ATLAS, a 512 × 512 single-photon avalanche diode (SPAD) array with embedded autocorrelation computation, implemented in 3D-stacked CMOS technology, suitable for single-photon correlation spectroscopy applications, including diffuse correlation spectroscopy (DCS). The shared per-macropixel SRAM architecture provides a 128 × 128 macropixel resolution, with parallel autocorrelation computation, with a minimum autocorrelation lag-time of 1 µs. We demonstrate the direct, on-chip computation of the autocorrelation function of the sensor, and its capability to resolve changes in decorrelation times typical of body tissue in real time, at long source-detector separations similar to those achieved by the current leading optical modalities for cerebral blood flow monitoring. Finally, we demonstrate the suitability for in-vivo measurements through cuff-occlusion and forehead cardiac signal measurements.

## 1. Introduction

Photon correlation spectroscopy (PCS) [1] is a statistical optics technique used to study molecular diffusion dynamics, determine the size distribution of small particles in suspensions, and measure non-invasive tissue perfusion. One such PCS technique is diffuse correlation spectroscopy (DCS), which has been used for monitoring the motion of scattering particles in tissue, particularly red blood cells (RBC). DCS has been investigated for applications such as oncology [2], the perfusion of skeletal muscle [3] and, in particular, the monitoring of cerebral blood flow [4–17], which is an important indicator of brain health and function [18].

Diffuse correlation spectroscopy [19] involves directing long coherence length light into a scattering medium, generating a speckle pattern from light that exits the medium, and capturing the intensity time series I(t) , of some part of this speckle pattern using a detector of suitable bandwidth. The autocorrelation of this time series, $g_{2}(\tau )$ , is then calculated according to Eq. (1), where τ is the autocorrelation lag time.

(1) $$g_{2}(\tau )=\;\frac{\left\langle I(t)I(t+\tau \rangle \right)}{{\left\langle I(t)\right\rangle }^{2}}$$

For spatially-coherent polarized chaotic light, $g_{2}(\tau )$ is related to the electric field autocorrelation $g_{1}(\tau )$ (Eq. (2)), by the Siegert relation (Eq. (3)).

(2) $$g_{1}(\tau )=\;\frac{\langle E^{*}(t)E(t+\tau )\rangle }{\left\langle I(t)\right\rangle }$$

(3) $$g_{2}(\tau )=1+\beta {\left|g_{1}(\tau )\right|}^{2}$$

Thus, the intensity autocorrelation $g_{2}(\tau )$ contains information on the fluctuations of the electric field due to the changing positions of scattering particles. A model that represents the expected motion of the scatterers, typically some form of stretched exponential function [20], is fitted to the autocorrelation, to provide some quantitative measure of the particle dynamics, such as relative changes in blood flow.

The decorrelation times due to the motion of scattering particles within tissue require that the autocorrelations are calculated with microsecond lag times. For monitoring of blood flow at depth, in order to ensure that the measurement contains information on changes within the deep tissue, rather than the superficial layers, large separations between the source and detection optics are needed [21,22]. Absorption and scattering over these large path lengths typically results in low photon numbers reaching the detector, making high signal-to-noise ratios (SNR) difficult to achieve.

Improvements in SNR can be found by using an array of single-photon detectors to measure a larger number of independent speckle modes from the speckle pattern in parallel. This approach, termed multispeckle DCS [23,24], has been shown to increase the SNR, but to date has required advanced hardware and processing capabilities. Previous attempts to use arrays for multispeckle DCS [24–34] have been limited by detector efficiency, pixel resolution, high data rate, and the need for external computing resources to process the data in real-time. To enable wearable systems with the potential for deep tissue measurements, real-time autocorrelation output is required, together with small form factor, high channel number and high detection efficiency [21].

To address these challenges, we present ATLAS, an “on-chip compute” 512 × 512 SPAD camera [35]. ATLAS uses advanced 3D-stacked backside illuminated (BSI) SPAD technology [36] to provide enhancements in detection efficiency and enable embedded computation of the autocorrelation function. While recent works [31,34] have demonstrated real-time autocorrelation processing capabilities using an external field programmable gate array (FPGA) connected to a general-purpose SPAD array, we present a custom-designed, large array SPAD camera tailored for multispeckle DCS applications. Each macropixel contains an entire autocorrelator in-pixel with a pitch of 40.68 µm. During each integration time, the macropixel calculates the $g_{2}(\tau )$ components directly, rather than streaming photon-counting frames off-pixel for each delay bin, minimizing the readout data transactions from the pixel to peripheral processing blocks. For multispeckle DCS applications, averaging of the individual macropixel autocorrelations is supported in an on-chip, high-speed “ensemble” mode. Among the advantages of this design over the FPGA-counterparts are shorter latency, higher data rate (not limited by FPGA connector), higher power-efficiency, and smaller form-factor.

In this article the design and operation of of the sensor is described and the autocorrelation imaging functionality is verified. The DCS measurement capability is also characterized, and the potential for monitoring of deep tissue blood flow in a multispeckle DCS system is demonstrated with in-vivo measurements.

## 2. Sensor architecture

**Fig. 1.**
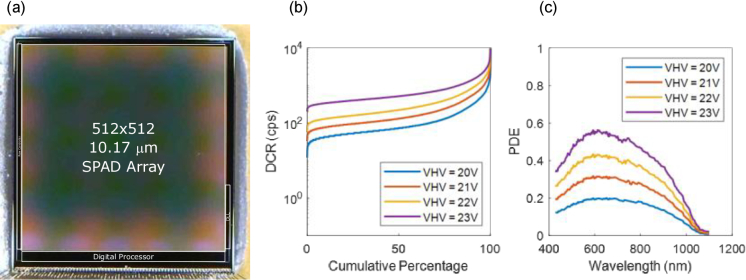
(a) Sensor chip micrograph. (b) Dark count rate per SPAD cumulative distribution. (c) Photon detection efficiency.

The sensor die photo is shown in Fig. 1(a). The top tier consists of a detector array of 512 × 512 of back-illuminated, 10.17 µm pitch deep trench isolated (DTI), micro-lensed SPADs in a 65 nm process technology. The array size has been selected to be comparable to existing SPAD sensors used for this application. The device has a one-sided bond-out allowing 3-side tiled arrays of sensors to be employed if larger areas are desired. Figure 1(b) and (c) also show the measured photon detection efficiency (PDE) and dark count rate (DCR) of the SPADs respectively. The PDE has been measured using the collimated output of a supercontinuum laser, with wavelength selection via an acousto-optic tunable filter, and shows a peak of 55% at 600 nm and 40% at 820 nm. A measurement at 820 nm with a source NA of 0.04, which is typical for diffuse correlation spectroscopy with this sensor, shows that the PDE is expected to be >85% of the value in collimated light. The standard deviation of PDE over the array is <10%. Median DCR is 500 Hz per SPAD at room temperature at a bias voltage of 23 V (breakdown voltage of 17.8 V) which differs from [36] due to our modified interface circuit. The dead time of the SPAD can be estimated from the maximum count rate (MCR) in [36] of between 113 Mcps and 51.8 Mcps to be between 3.2 and 7.2 ns. ATLAS can count photons to a maximum rate of 32 Mcps. In practical physiological DCS experiments we expect to operate in the region of 1 Mcps to 100 kcps and so a decade away from the sensor MCR and over two decades from the SPAD MCR.

**Fig. 2.**
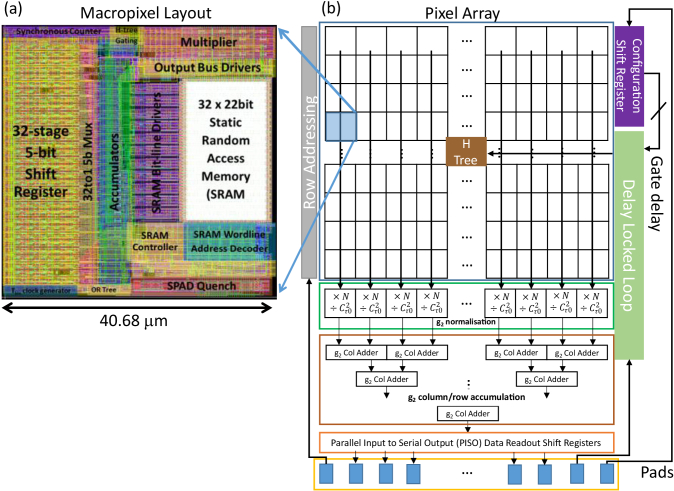
(a) Macropixel layout and (b) Sensor architecture showing the column normalization processor which multiplies each macropixel autocorrelation sample $A_{\tau }$ by the number of BinClk cycles (*N*) and divides by the square of the total photon count ${\left(C_{\tau 0}\right)}^{2}$ before summing each entire row in a pipelined adder.

The passively quenched SPADs are connected in groups of 4 × 4 through an OR-tree arrangement to 128 × 128 macropixels in the bottom tier 40 nm process arranged at 40.68 µm pitch (Fig. 2). Each macropixel is capable of computing a 31-tap autocorrelation function, $g_{2}(\tau )$ , of the time course of the intensity I(t) , as per Eq. (1), with a minimum correlation lag time, τ , of 1 µs.

**Fig. 3.**
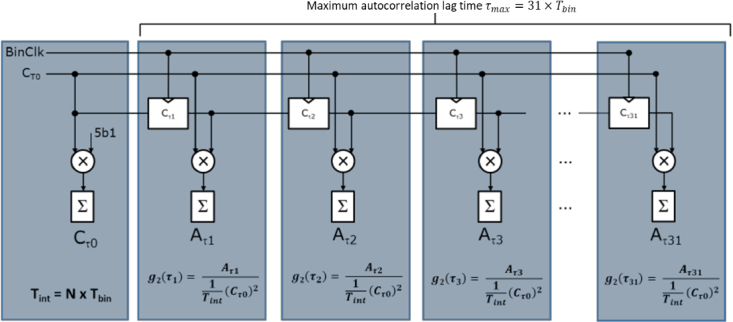
Macropixel signal flow diagram. 5-bit photon counts $C_{\tau 0}$ from the SPAD are delayed progressively 31 times, multiplied by the current value of $C_{\tau i}$ and accumulated as $A_{\tau }$ *.* The autocorrelation calculation of $g_{2}(\tau )$ needs to be normalized by ${\left(C_{\tau 0}\right)}^{2}/T_{int}$ .

The macropixel (Fig. 2(a)) is area efficient, using a 32 × 5-bit shift register implemented with custom D-type flip-flops and a shared Multiply-Accumulate (MAC) unit operating around a 32 × 22-bit high-density SRAM macro. To manage the high data rate and number of pixels while maintaining a high temporal aperture ratio and high sensor fill factor, various autocorrelation compression techniques were explored. A comparison revealed a linear version of the parallel autocorrelation FPGA algorithm proposed in [34,37] as the most fitting solution to the requirements. The hardware implementation of this algorithm is based on the signal flow diagram in Fig. 3. This implementation requires progressive delaying, multiplication and accumulation of photon counts and subsequent normalization to generate the $g_{2}(\tau_{i})$ , autocorrelation bins. The normalization operation is performed by a column parallel processor (shown at the bottom of Fig. 2(b)). This processor calculates and divides by the squared total photon count $C_{\tau 0}$ and multiplies by number of autocorrelation cycles *N* ready for scaling off-chip by the *BinClk* period $(T_{bin})$ . *N* is an integer representation of the total integration time $T_{int}$ (Fig. 3) which is equal to *N* times $T_{bin}$ .

**Fig. 4.**
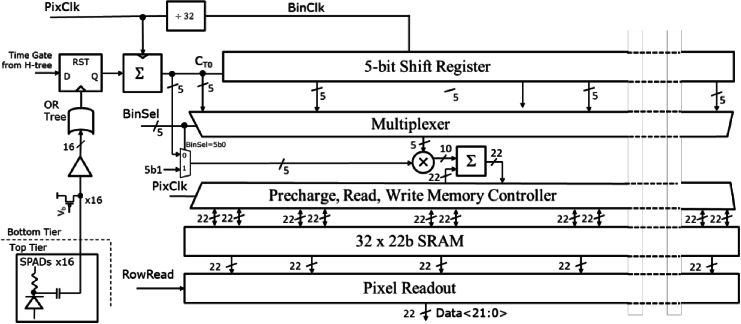
Macropixel circuit block diagram. This shows the hardware implementation of Fig. 3. 16 SPADs are OR-ed and counted in a 5-bit accumulator. A 31 stage 5-bit shift register creates delayed photon counts at *BinClk* rate. A shared multiplier multiplexed operating at 32 times higher frequency (*PixClk*) generates the $A_{\tau }$ samples in an 32 × 22b SRAM.

Figure 4 shows the macropixel block diagram. The SPAD front end is capacitively coupled from the top tier via a metal-oxide-metal capacitor and polysilicon resistor. This arrangement does not permit time gating of the SPAD or power-saving at high photon fluxes and the RC recharge time sets the dead time. The 16 SPAD outputs are rebuffered and fed into an OR-tree whose output latches a gate window pulse from an H-tree. If the gate window is high on the rising edge of the SPAD OR-tree event, the 5-bit photon counter will be incremented. This count enters the shift register every *BinClk* period and is reset at the beginning of each new 32-*PixClk* cycle.

**Fig. 5.**
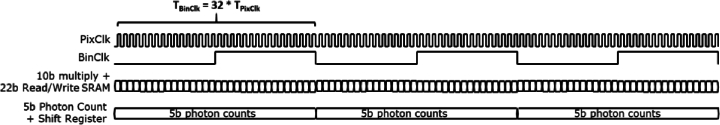
Macropixel timing diagram. In each BinClk period photon counts are delayed and shifted one place in the 5-b shift register. In that time *PixClk* initiates 32 10-b multiply and add (precharge, modify write) operations around each word location in the 32 × 22b SRAM.

The 31-tap 5-bit shift register is clocked 32 times slower than the time-multiplexed MAC unit which multiplies each delayed sample by the 5-bit photon count to generate a 10-bit result (Fig. 5). This is then summed with the corresponding 22-bit autocorrelation bin read out and written back to the SRAM bank. 32 such operations are performed within one cycle of photon counting and shift.

The macropixel is clocked at a maximum *PixClk* rate of 30 MHz (limited by current PCB I/O but designed for 100 MHz operation). The macropixel completes the 32, 5-bit shift, 5-bit multiply and 22-bit summation and SRAM read/write operations within the 1 µs *BinClk* correlation lag time. For the entire macropixel array this employs 120 M transistors and applies to 0.5 T MAC operations per second (Op/s) and consumes around 0.3 W power.

To summarize, the output of the sensor in autocorrelation imaging mode is a 128 × 128 array of 32 values. The first of these values is the number of photon counts during the integration time, and the remaining 31 values correspond to the 1^st^ to 31^st^ lag times of a biased autocorrelation. The autocorrelation is of a time-series of length *M*, where *M* is the number of iterations of the 32- *PixClk* cycle. *M* is thus equal to the number of clock cycles during the integration time divided by 32 and the lag time is equal to 32 divided by *PixClk*.

A conventional rolling row-addressing scheme reads the macropixel autocorrelation data to a synthesized digital processor unit to normalize and sum the information to enable high SNR measurements with greatly reduced I/O bandwidth requirements (164-bytes per frame). A column-parallel normalizer block divides the 31 $g_{2}(\tau_{i})$ , autocorrelation taps by ${\left(C_{\tau 0}\right)}^{2}/T_{int}$ as they are read sequentially from the currently addressed macropixel row SRAMs. The divider takes 27 *PixClk* cycles to generate the 27-bit result. The divider array has 4 × 128 individual divider channels to schedule and overlap individual bin division operations to reduce normalization and readout time. This normalized data is fed to a combinatorial adder tree which sums the 128 bins (*A_τn_*) across a row, followed by an accumulator which accumulates the row sums, calculating ensemble average autocorrelation and total photon count for the whole array. The final output data is a 41-bit global photon count and a 31-bin, 41-bit depth fixed-point representation autocorrelation sequence.

This on-chip computation combined with a single-side bond out is foreseen to allow a 3-side buttable arrangement of sensors with shared control signals from a microcontroller or FPGA for future wearable, low-cost DCS systems. Data is read from a 16-bit I/O bus at 30 MHz and the chip operation is configured via a serial interface. In the current implementation an FPGA with a USB3 interface is used to provide the necessary clocks, set required parameters and read the data from the SPAD array.

Table 1 compares our device with other SPAD sensors which have been used for DCS but without on-chip autocorrelation computation. We achieve over 50x PDE at optimal tissue penetration wavelengths due to our backside illuminated technology whilst providing one of the highest pixel ensemble-averaging SNR gains at this autocorrelation lag time. Front-side illuminated sensors can also employ microlenses [38] although they usually start from a lower fill-factor than comparable back-side illuminated SPADs requiring a high concentration factor and therefore greater angular dependence, sensitivity to manufacturing alignment and variability. An example is the sensor in [39] which has a pixel pitch of 16.38 µm and a native fill factor of 10.5% and employs die-level microlens to attain a concentration factor up to 4.2. At 785 nm this will result in a maximal PDE of around 6.2%. ATLAS employs wafer-scale microlens with PDE of 50% at maximum excess bias and excellent uniformity through precise foundry-wafer alignment processes.

**Table 1.** Table of comparison of SPAD arrays used for DCS

Paper ID	This work	[31]	[25]	[34]
Technology	65 nm/40nm	180nm	130nm	40nm
	BSI Stacked	FSI^*a*^	FSI	FSI
Correlation Computation	On-chip	FPGA/software	software	FPGA
Pitch (µm)	40.68	16.38	50	9.2 × 19.4
SPADs/Macropixels	512 × 512/128 × 128	512 × 512	32 × 32	192 × 128
Fill Factor (%)	100	10.6	1.5	13
Max PDE @ 785 nm (%)	47	1.48	0.12	1.04
Min. lag time (µs)	1	0.38	1.4	38.4
Pixels at minimum lag	16384	8000	1024	24576
SNR gain min. lag	117	89.1	∼32	110
DCS frame rate (fps)	40	17	Off-line	16.7
Ensemble DCS rate (fps)	27000	17	Off-line	16.7
Sensor Power (mW)	300	700^*b*^	80	30

^*a*^ Front side illuminated.

^*b*^ power consumption of [31] is estimated from a sensor in [38] with similar array size and single bit readout, reported in bright conditions as 700 mW.

The sensor also enables several auxiliary operating modes which are useful for initial alignment and characterization or alternative applications such as time-domain DCS, high dynamic range global shutter and time gated fluorescence lifetime imaging. These include (1) 1.2 kfps rolling shutter 22-bit single photon counting and (2) time-gated global shutter 22-bit photon counting via a balanced H-tree (300ps minimum gate duration). Time gates may be generated by the FPGA or delay locked loop (DLL) with 11-30 ps granularity, (3) raw and normalized per-macropixel 31 tap correlation imaging at 41 fps, and (4) ensemble average of 16384 macropixel 31-bin autocorrelation at 27 kfps.

## 3. Sensor verification

### 3.1 Verification of autocorrelation computation

**Fig. 6.**
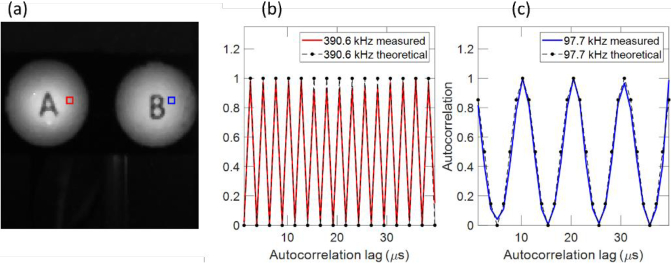
(a) Target for verification of autocorrelation imaging mode. Example autocorrelations from the highlighted pixels in (a), corresponding to frequencies of 390.6 kHz (b) and 97.7 kHz (c).

**Fig. 7.**
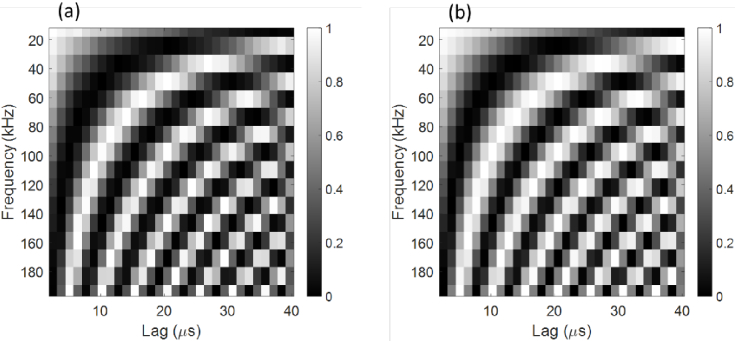
(a) Measured and (b) theoretical normalized autocorrelations for frequencies between 12.2 and 195.3 kHz.

To verify the correctness of the on-chip autocorrelation calculation, and that the SNR increases as expected with the increasing number of pixels, modulated light sources were used, and the measured autocorrelations compared to the theoretical values. The autocorrelations were corrected for bias by multiplying the value of the $n^{th}$ lag by M/(M−n) , where *M* is the number of iterations. For the autocorrelation imaging mode *PixClk* was set at 25 MHz and two diffusers with printed text overlayed, were illuminated by two red LEDs (Fig. 6(a)), and a 25-mm focal length lens was used to image the diffusers onto the sensor. The LEDs were forward biased by a dual-channel waveform generator (Keysight 33500B), and independently modulated with sinusoidal waveforms between 12.2 and 390.6 kHz (the maximum non-aliased frequency at *PixClk* = 25 MHz), and rectangular pulse waveforms between 1 kHz and 195 kHz. In contrast to intensity images, timing characteristics of these modulated waveforms can be acquired directly from macropixel autocorrelation signals. Figure 6(b) and (c) show examples of measured and theoretical normalized autocorrelations, from macropixel (45, 49), located in the ‘A’ target and illuminated with a frequency of 390.6 kHz, and macropixel (111, 49) located in the ‘B’ target and illuminated with a frequency of 97.7 kHz. Figure 7 demonstrates that there is excellent correspondence between measured (a) and theoretical (b) autocorrelations for 16 example frequencies between 12.2 and 195.3 kHz, in steps of 12.2 kHz. Sinusoidal measurements were carried out using 8192 BinClk cycles corresponding to an interval of 10.49 ms.

**Fig. 8.**
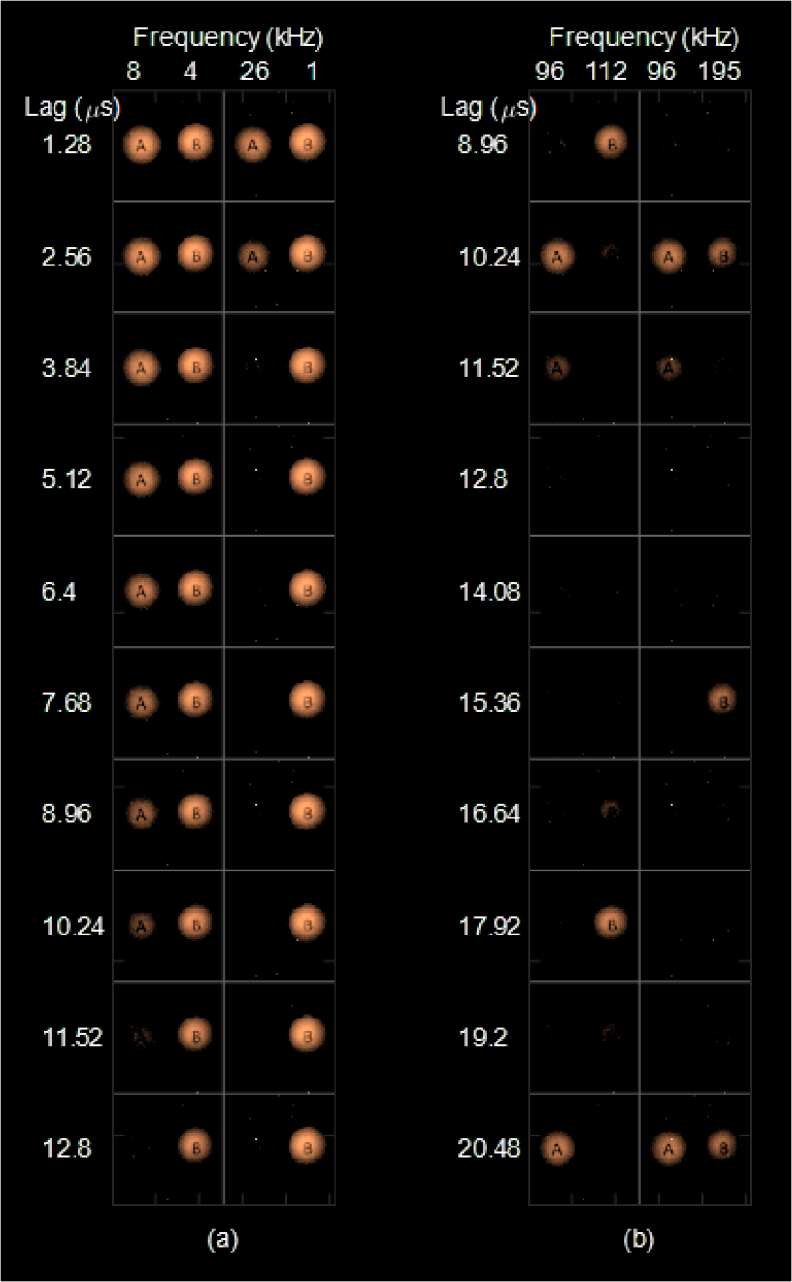
Autocorrelation image sequences with 10% duty cycle pulsed wave LED illumination of A and B targets. (a) Low frequency 8 kHz/4 kHz and 26 kHz/1 kHz pulsed wave images over a 1.28-12.8 µs lag range. (b) High frequency 96 kHz/112 kHz and 96 kHz/195 kHz pulsed wave images over an 8.96-20.48 µs lag range.

Autocorrelation image sequences with 10% duty cycle pulsed wave LED illumination of A and B targets are shown in Fig. 8. Rectangular pulse images were captured using 2048 BinClk cycles (2.62 ms). These sequences highlight different autocorrelation image signatures at low and high frequencies, together with the capability to isolate one image signature from another through appropriate selection of autocorrelation lags and modulation frequencies. Figure 8(a) shows low frequency 8 kHz/4 kHz and 26 kHz/1 kHz pulsed wave images over a 1.28-12.8 µs lag range. Figure 8(b) shows high frequency 96 kHz/112 kHz and 96 kHz/195 kHz pulsed wave images over an 8.96-20.48 µs lag range. Two characteristic time-scales of pulsed waveforms can be identified through autocorrelation - the pulse-high duration and the pulse period. The smallest resolvable lag interval is 1.28 µs. Autocorrelations of 10% duty cycle 1 kHz and 4 kHz pulsed waves (B targets) vary slowly over the lag range. At 10% duty cycle 8kHz (A target), pulse-high decorrelation occurs by 12.5 µs causing target A to gradually disappear from the sequence. At 10% duty cycle 26 kHz (A target) pulse-high decorrelation occurs by 3.84 ms. At 96 kHz, 112 kHz and 195 kHz the pulse period occurs on lag intervals of 10.4 µs, 8.9 µs and 5.1 µs respectively. Both A and B targets disappear from the high frequency autocorrelation image sequence at lag positions not corresponding to the pulse period.

**Fig. 9.**
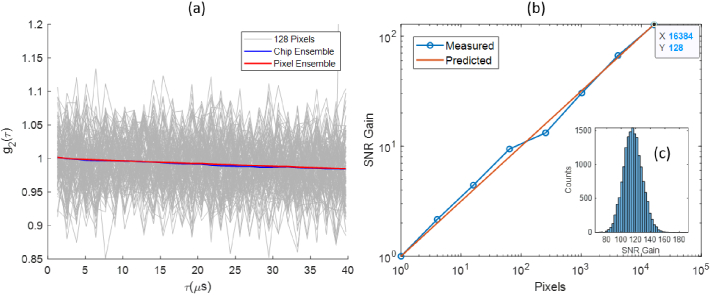
(a) Ensemble average correlation calculated on-chip (blue) and off-chip (red). (b) Ensemble average SNR gain with respect to the single macropixel mean SNR at increasing number of pixels.

Repeated frame acquisition with uniform illumination with a single LED at DC was used to characterize DCS SNR. To demonstrate that ensemble mode is functioning as expected, Fig. 9(a) compares on-chip ensemble mode DCS values (blue) with averaged DCS values (red) computed off-chip from 16384 individual macropixel DCS results. Normalized autocorrelation traces for 128 separate macropixel (in grey) are also shown to indicate the variance. Figure 9(b) demonstrates that the DCS SNR gain of the array, relative to macropixel (64,64), as a function of the number of macropixels increases close to a square root basis as predicted by theory [24]. SNR gain is a distribution when measured as a ratio of the all-array SNR to the individual SNR values of all single macropixels as shown in Fig. 9(c). The mean all-array SNR gain is measured to be 117 with a standard deviation of +/- 26. The variance in Fig. 9(c) can be accounted for in part by the interaction of macropixel microlens numerical apertures with LED illumination conditions.

### 3.2 Accuracy of time constant measurement for diffuse correlation spectroscopy

To assess the sensor's ability to accurately measure time constants from exponentially decaying autocorrelations, the ATLAS sensor was illuminated with a high-bandwidth LED, modulated by the Keysight 33500B waveform generator to produce autocorrelations with exponential decay. As per [40] the recurrence relation shown in Eq. (4) was used to generate suitable LED intensity sequences of length 64000. In this recurrence relation $f=e^{-1/(\nu \tau_{c})}$ where ν is the output sample rate and $\tau_{c}$ is the time constant, $G_{n}$ is a pseudorandom number drawn from the standard normal distribution and $I_{0}=G_{0}$

(4) $$I_{n}=fI_{n-1}+\;G_{n}\sqrt{1-f^{2}}\;\text{for}\;n>0$$

**Fig. 10.**
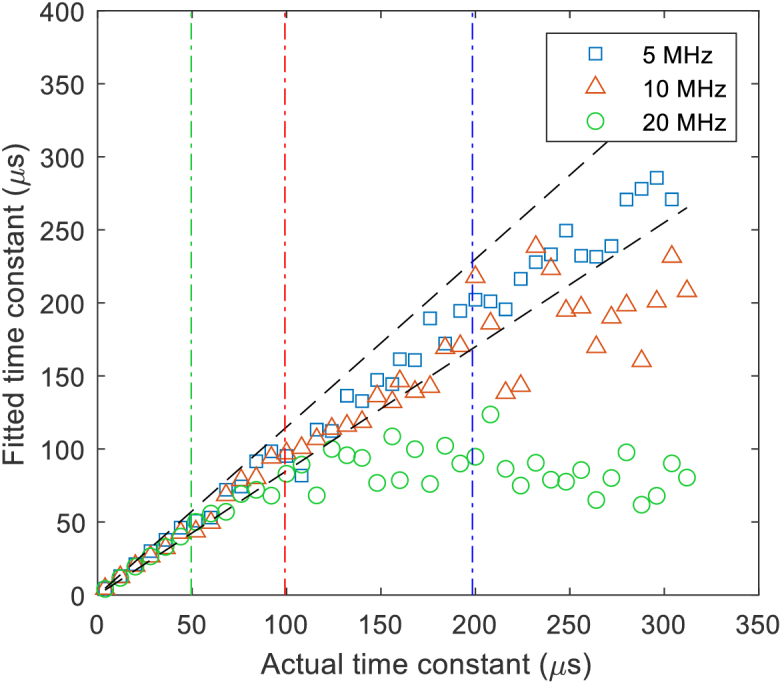
Time constants from exponential fitting of autocorrelation of LED sequences.

The correctness of these sequences was confirmed by calculating the 64000-lag unbiased autocorrelation of the LED intensity acquired using a high-speed photodetector (Thorlabs DET10C2) and oscilloscope, and fitting an exponential decay to the result. LED sequences resulting in time constants of 4 to 156 µs and 160 to 312 µs in steps of 8 µs were measured using ATLAS with 8192 iterations and *PixClk* values of 5, 10 and 20 MHz. The measured autocorrelations were corrected for bias and fitted with an exponential function. Values for the fitted time constant are shown in Fig. 10, the colored dashed-dotted vertical lines show the maximum lag for *PixClk* values of 20, 10 and 5 MHz, the measured values are typically within 15% of the actual values (black dashed lines) when the time constant is less than 1.5 times the maximum lag time.

### 3.3 Sensitivity limit for diffuse correlation spectroscopy

**Fig. 11.**
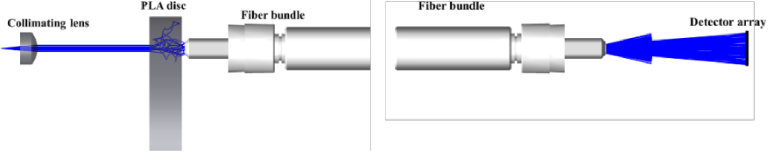
Illustration of experimental setup to assess the sensitivity for DCS measurements.

To measure the sensitivity limit of the ATLAS sensor for DCS, we have used the setup in Fig. 11. A stabilized laser diode (Thorlabs LP785-SAV50) was driven in continuous wave mode using a MLD203CLNE low-noise constant current driver, to give a (driver-limited) maximum optical output power of 20 mW. The laser was collimated to a 1-mm diameter beam using a F230APC-780 collimation optic, and attenuated as necessary by adjusting the drive current and with neutral density (ND) filters. The beam was then made incident 4 mm from the edge of a 5 mm thick, 40 mm diameter disc of white polylactic acid (PLA). The PLA disc was rotated about its center, using a DC motor driven with a square wave profile at 1 Hz. Light transmitted through the disc was collected with a fiber bundle, (Thorlabs BF20LSMA01), consisting of seven 550-µm core multimode fibers, with an effective core diameter of 1.75 mm and an NA of 0.22, and relayed to the detector array.

**Fig. 12.**
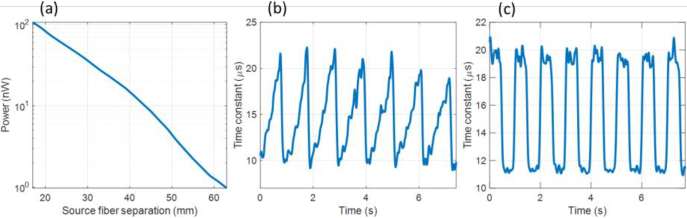
(a) Measured optical power from the end of the detector fiber bundle at different source-detector separations on human forehead. (b) Typical range of time constants measured from adult forehead from exponential fitting of autocorrelations acquired with ATLAS in ensemble mode, with a 10 mm source detector separation and an integration time of 13.1 ms (8192 iterations) per sample. (c) Time series of time constant from exponential fitting of autocorrelations acquired with ATLAS in ensemble mode, from a rotating PLA disc (Fig. 11), driven with a square wave voltage to produce a similar range of time constants as measured from the forehead.

To understand the typical powers available for DCS with this system, the power exiting human tissue has been measured at several distances from the point of incidence of the light. An 820 nm continuous wave laser diode (L820P100), with 34 mW of power (the maximum permissible exposure (MPE) at this wavelength [41]) was used as the light source. This source was focused to a 1 mm diameter spot using an 8 mm focal length, 0.5 NA lens (Thorlabs A240TM-B) and directed at an adult subject’s forehead. The light exiting the forehead was collected using the detector fiber bundle and the total power at the distal end of the detector fiber was measured using a photodiode power sensor (Thorlabs S130C). Figure 12(a) shows the measured optical power at several source-detector separations (the distance between the incident light and the proximal end of the detector fiber) between 17 and 63 mm. For example, the measured optical power at 43.5 mm source-detector separation was 10 nW.

To understand typical decorrelation timescales for tissue measurements, the DCS system in ensemble mode was used to measure the intensity autocorrelation from an adult subject’s forehead. For these measurements *PixClk* was set at 20 MHz, to give 1.6 µs lag time, and 8192 iterations were used to give an integration time of 13.1 ms per sample. A 200 µm fiber was used to collect the light and relay it to the detector, with a speckle-to-macropixel size ratio of unity. The separation between the source and the proximal end of the fiber was 10 mm. Figure 12(b) shows the typical range of the time-constant from exponential fitting of the bias-corrected autocorrelation curves (average R^2^ = 0.98). The minimum and maximum time-constants measured over this timescale were 9 µs and 22 µs respectively. To mimic this range, the PLA disc was rotated such that the time constants, measured using the same DCS system and exponential fitting (average R^2^ = 0.99), approached a square-wave values between 11 µs and 20 µs (Fig. 12(c)).

**Fig. 13.**
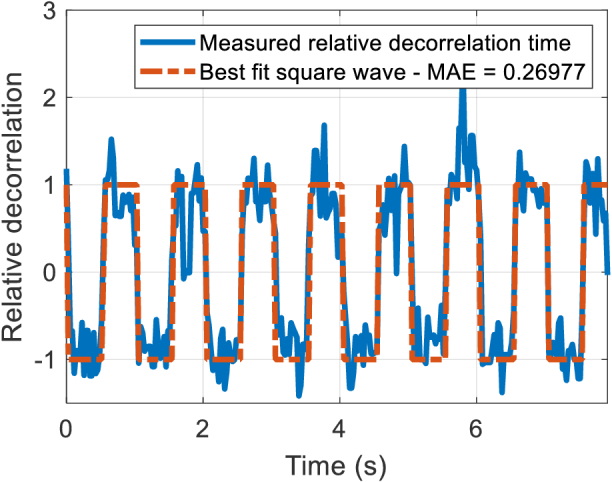
Normalized time series of relative time constant from exponential fit, and best fit square wave.

To assess the fidelity of the DCS measurements for different powers from the fiber, the disc was illuminated with decreasing laser intensity, beginning with a level that resulted in 30 nW total power at the distal end of the fiber bundle and decreasing to 3 nW. The time-constant from an exponential fit to the bias-corrected autocorrelation was used as the relative decorrelation time. The DC component of the time series of relative decorrelation times was subtracted, and the result normalized to a mean amplitude of 1. A 1 Hz square wave was then fitted to this time series and the mean absolute error (MAE) between the decorrelation time series and the square wave was used as the figure of merit (Fig. 13). An MAE of 0.9 or above indicates that the measured time series is essentially random.

A key parameter to optimize is the average speckle diameter at the detector plane, which is related to the separation, *z*, between the distal end of the fiber-bundle and the detector array, as per Eq. (5).

(5) $$\frac{\lambda z}{a}$$

where λ is the wavelength and *a* is the maximum transverse extent of the fiber core. Nyquist sampling of the speckle maximizes the speckle contrast, however the intensity of the light on the detector is inversely proportional to $z^{2}$ , leading to a tradeoff between speckle contrast and photon counts. To determine the optimal separation between the detector and distal end of the fiber, the separation has been scanned using a motorized linear translation stage during measurement of the rotating disc. The optimal separation was chosen by the lowest MAE and is found to be 23 mm. From Eq. (5) (and verified with a Huygens simulation of the fiber bundle in Ansys Zemax OpticStudio) this separation corresponds to a speckle size of 10.3 µm, which is smaller than the macropixel size of 40.68 µm, but close to the SPAD size of 10.17 µm.

**Fig. 14.**
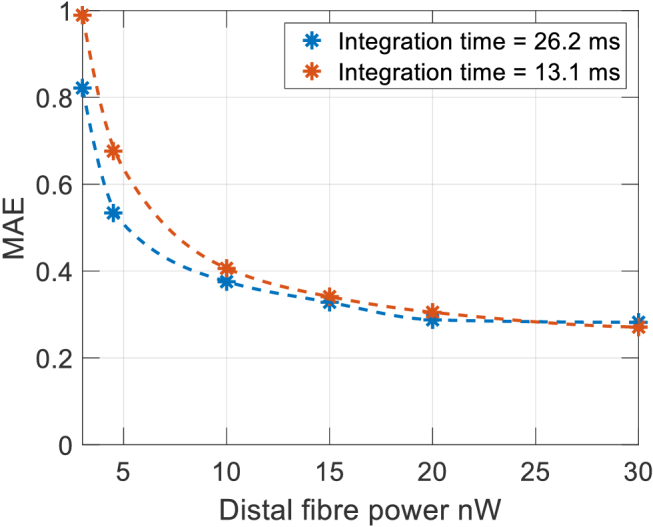
MAE against distal fiber powers between 3 and 30 nW.

Plots of MAE against power from the distal end of the fiber, for integration times of 13.1 ms (8192 iterations) and 26.2 ms (16384 iterations) are shown in Fig. 14. When the power from the fiber is below 10 nW the MAE begins to rise rapidly. Some detectable signal remains at 4.5 nW which, from Fig. 12, corresponds approximately to a source to detector separation of 50 mm at the forehead.

## 4. In-vivo measurements

To demonstrate the suitability of the sensor for in-vivo measurements we have conducted a cuff occlusion measurement, and measurements of the cardiac signal from the forehead of an adult subject.

### 4.1 Cuff occlusion measurement

**Fig. 15.**
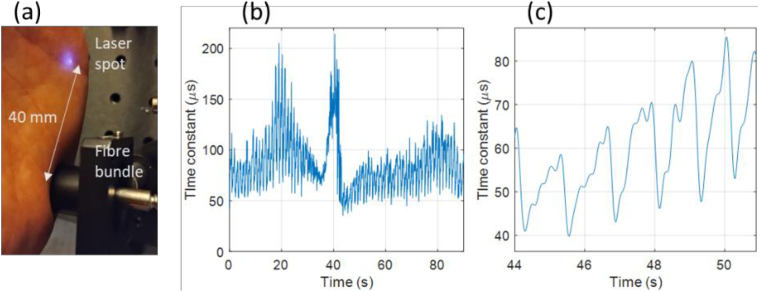
(a) Source and detection fiber at palm. (b) Time constant during and after a linear increase of wrist occlusion pressure from 0 mm Hg to a peak of 165 mm Hg at 40 s. (c) Six pulse periods of the time constant post occlusion.

To demonstrate the ability of the sensor in resolving changes in blood flow, DCS measurements were acquired in ensemble mode. A *PixClk* of 5 MHz was used with an integration time of 13.1 ms per sample (2048 iterations, with a sample rate of 45 Hz). The measurements were taken at the ulnar side of the left palm (Fig. 15(a)), with a laser to detection fiber separation of 40 mm. Measurements were taken during and after a gradual, approximately linear increase of occlusion pressure of the left wrist, starting at 0 mm Hg and reaching a maximum of pressure of 165 mm Hg at 40 s, at which point there is an abrupt decrease to 0 mm Hg. Figure 15(b) shows a plot of the time constant from exponential fitting of the autocorrelation values (after bias correction) over this time, low-pass filtered with a cut-off frequency of 5 Hz. The time series shows the characteristic post-occlusion reactive hyperemia. Also shown Fig. 15(c)) is an example of six periods of the pulse waveform post occlusion. The source used for these measurements was the same low-cost single-mode 820 nm laser diode (L820P100) used in Section 3.3. This laser diode is of a type that has previously been shown to be suitable for DCS [42], and was focused to a 1 mm diameter spot, with an optical power of 34 mW.

### 4.2 Cardiac signal from forehead

**Fig. 16.**
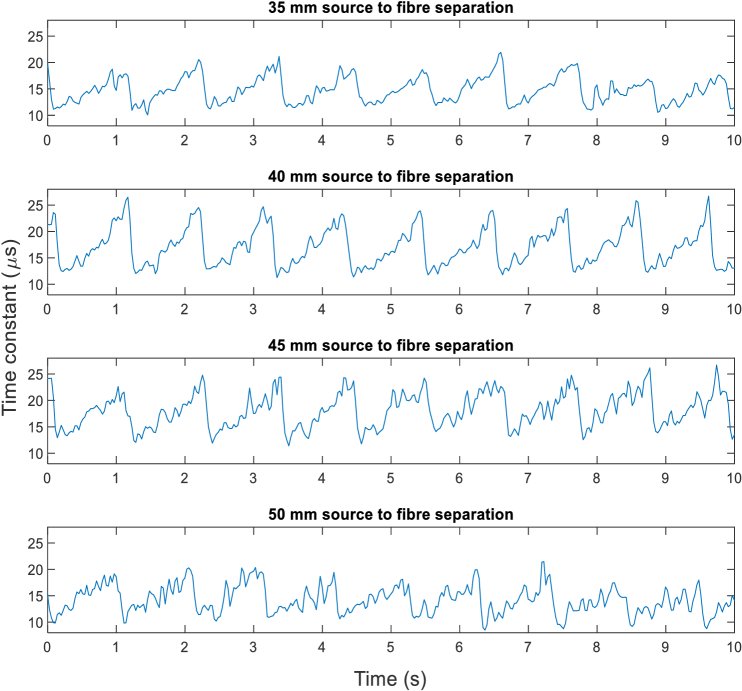
Time constants from exponential fitting of autocorrelations measured from forehead, for separations between the source and fiber of 35, 40, 45 and 50 mm.

Figure 16 shows the time series of time constants from exponential fitting (after bias correction) of ensemble mode DCS measurements (with *PixClk* = 20 MHz) of the cardiac signal from a forehead of an adult subject. The L820P100 laser diode was used as the source, with source to fiber separations of 35, 40, 45 and 50 mm. An integration time of 26.2 ms (16384 iterations) was used with a sample rate of 35 Hz. The results are in agreement with the results shown in Fig. 12(a) and Fig. 14 and are comparable to the largest source detector separations reported to date [12,32,33,43].

## 5. Discussion

We have demonstrated a 512 × 512 SPAD array with 128 × 128 macropixels, each with embedded autocorrelation computation, operating at clock rates to 30 MHz. This array can output autocorrelation images, or an average autocorrelation for all pixels, with lag times down to 1 µs.

Off-chip computation of autocorrelation functions for DCS from SPAD imagers provides full access to speckle information and unlimited time history from which to derive autocorrelation functions over many decades of blood flow time constant [31]. This is of great value in providing a reference scientific instrument on which to base biomedical studies and deeper understanding of physiological effects. This nevertheless necessitates a tradeoff in the SPAD imager between the number of pixels, minimum time constant and I/O rate of the sensor which mean that the full-resolution of the array is not accessible at the fastest sampling rates [31]. Moreover, these systems involve high power-consumption in both the sensor I/O bus as well as within the FPGA autocorrelator, and are relatively bulky and not amenable to wearable formats. Embedding the autocorrelator with the sensor indeed breaks the I/O bottleneck between sensor and FPGA allowing parallel computation over the full array at the shortest lag times. On-chip accumulation of the per-macropixel autocorrelations offers vastly reduced data rates and hence I/O power. In ATLAS, there is a compromise made in reduced range of lag times that can be captured in a single frame due to the limited area within the macropixel for the autocorrelator processing. However, our LED emulation results show that, provided the blood flow time constant is within the maximum lag time range, there is little loss of accuracy versus exploiting the full temporal record. ATLAS sensors can also be 3-side tiled to further increase SNR without compromise on minimum blood flow time constant. The embedded computation is the enabling factor which allows modest hardware support requirements (microcontrollers) and small printed circuit board formats providing a pathway to multiplexed, large area, wearable formats. Note that in 3D-stacked BSI sensor implementation, no compromise needs to be made on detector performance. Higher NIR photon detection efficiency is readily obtained which maximizes the SNR and source detector separation within the maximum permissible exposure at these wavelengths. FPGA-based correlators often employ a multi-tau approach allowing logarithmically spaced autocorrelation [37]. This requires multiple clocks and accumulators between shift registers crossing clock boundaries. FPGAs are relatively unconstrained in computational resources compared to custom 3D stacked image sensors where the autocorrelator circuitry must be placed under the SPAD detectors. In ATLAS, we chose to implement a linearly spaced autocorrelator to manage design complexity and minimize macropixel area. Multi-tau type functionality is possible by taking multiple frames at progressively reduced clock rates and stitching the resulting autocorrelation functions together off-chip at the expense of frame rate.

The implementation of autocorrelation functionality at the macropixel level enables real-time calculation of the autocorrelation function, which is essential for practical DCS measurements. Such “on-chip compute” SPAD arrays are necessary to enable large array and MHz frame rate multispeckle DCS systems, which could lead to >1Tbit/sec data rate. Our results show that the autocorrelation imaging functionality works as intended, as validated by the imaging of targets illuminated with modulated LEDs.

The suitability of our SPAD array for in-vivo measurements has been demonstrated through a cuff occlusion experiment, to show sensitivity to changes in blood flow that are observed during various physiological conditions. The ability to measure the cardiac signal at the forehead with large source-detector separations highlights the potential of this sensor for non-invasive monitoring of cerebral blood flow. The real-time calculation of the autocorrelation function, eliminates the need for post-processing and reducing computational burden. This enables faster data acquisition rates, and opens up the possibility for tiling arrays to further improve SNR, and for simultaneous measurements at multiple locations. The compact size and low power consumption of the detector at low intensities make it an attractive option for wearable or portable devices. Note that the power reported in Table 1 of 300 mW is for continuous operation of the ATLAS sensor at maximum clock rate. For wearable operation we foresee using lower clock rates (e.g. 5 MHz) to match the autocorrelator lag range around typical blood flow time constants. Further power savings can be achieved by clock duty cycling to capture the waveform at significant points on the cardiac waveform [44]. This could enable continuous monitoring of cerebral blood flow in various settings, such as during exercise, sleep, or in patients with neurological disorders. Future studies can build upon this work by exploring the application of our SPAD array for measurement in patients with cerebrovascular disease or traumatic brain injury. Combining such a system with other optical modalities, such as near-infrared spectroscopy (NIRS) [15] and speckle contrast optical spectroscopy (SCOS) [12], could provide a more comprehensive understanding of cerebral hemodynamics.

The autocorrelation imaging functionality may be suitable for other photon correlation spectroscopy applications such as laser speckle rheology [45] and particle sizing and analysis [46]. Other potential applications are found in microscopy, such as fluorescence correlation spectroscopy (FCS) [47] and single-molecule-localization-microscopy (SMLM) [48,49]. The high-speed photon counting capability has potential applications in spinning disk confocal microscopy and microfluidics.

## Data Availability

Data underlying the results presented in this paper are available in Ref. [50]
